# Healing Relationships: A Qualitative Study of Healers and Their Clients in Germany

**DOI:** 10.1155/2015/145154

**Published:** 2015-06-07

**Authors:** B. M. H. Stöckigt, F. Besch, F. Jeserich, C. Holmberg, C. M. Witt, M. Teut

**Affiliations:** ^1^Institute for Social Medicine, Epidemiology, and Health Economics, Charité-Universitätsmedizin Berlin, 10117 Berlin, Germany; ^2^Catholic Academy “Die Wolfsburg”, Ruhr, 45478 Muelheim, Germany; ^3^Berlin School of Public Health, Charité-Universitätsmedizin Berlin, 13347 Berlin, Germany; ^4^Institute for Complementary and Integrative Medicine, University of Zurich and University Hospital Zurich, 8091 Zurich, Switzerland

## Abstract

*Background*. The aim of this study was to investigate the nature of the relationships between healers and their clients in Germany. *Methods*. An interdisciplinary research team performed semistructured interviews with healers and clients and participatory observation of healing sessions. All interviews were digitally recorded, transcribed, and analyzed using content analysis. *Results*. Fifteen healers and sixteen clients were included. The healer-client relationship was described as a profound and unique experience, which brought forth interpersonal and spiritual connections. The healers were seen as role models for healing to occur and support for being connected spiritually. The clients had to be open-minded and responsible. The importance of the healers' empathy was emphasized. *Discussion*. The relationship between healer and client can be seen as a triangular connection between client, healer, and a transcendent source which is not the case in typical patient-doctor relationships. The spiritual connection is also said to enhance the empathetic understanding of the healer. The personality and a partner-like attitude of the healer supported the client in giving a more positive meaning to his life, in reconnecting to his spirituality, and in taking responsibility. Future studies should address the role of spirituality in health care and the development of enduring healer-client relationships.

## 1. Introduction

The relationships between health care providers and health seekers are a topic of interest in various disciplines. Historically, the term “therapeutic relationship” derives from psychotherapy and is described as a special form of interaction between therapist and client. Beside the therapeutic method used, the therapeutic relationship is said to have an effect on its own on the outcome of the therapy [1]. Rogers, one of the first dealing intensively with this subject, argued that core conditions of the therapist in the therapeutic relationship are congruence, unconditional positive regard, and empathetic understanding [2]. Over the last decades, the model of the therapeutic relationship has been further developed to include the importance of the clients' part in the relationship, whose early conceptualizations of the therapeutic relationship failed to emphasize [3, 4].

In medical research, the doctor-patient relationship was historically doctor-centered [5]. Today, many patients in a technology-oriented medical system expect a holistic and empathetic approach of their doctors, wherein communication plays an important role and an active part in therapy for them [6–9]. A recent study found that an empathic doctor-patient relationship fosters better compliance, more satisfaction, and better health outcomes among patients and more satisfaction and less frequent burn-out among doctors [10].

Anthropological research, too, highlights the therapeutic impact of the relationship between healers and clients in ritual healing. Therein, the personality of the healer plays an important role [11–16]. Before becoming healers, many healers experienced a spiritual transformation which (they believe) empowered them with the capability to heal. Koss-Chioino talks in this regard of “radical empathy,” which means that the healer can connect with the experiences and feelings of the client in the healing ritual [17]. Additionally, the personality of the healer and the symbolic and metaphoric aspects of healing rituals which give meaning to the illness in the cultural context are thought to have high impact on the activation of resources, health maintenance, and outcomes [11–15, 18–20].

Though all these disciplines put special emphasis on certain, slightly divergent aspects of the relationship, we can infer that the interaction between doctor/healer/therapist and patient/client is considered crucial for the healing/therapeutic process [21] and that the creation of meaning and compliance within this relationship is considered to have a strong significance [22].

In Germany, some healing techniques (e.g., laying on of hands) have a long cultural-historical tradition [23]. In the 1990s, the number of at least 7000 healers was estimated [24]. Today, the total number of healers is unknown but may have increased considering the positive trend of CAM usage in the last decades [25]. Healers' practices and backgrounds have become more and more diverse which may be related to influences by globalization and new age [23, 24, 26, 27]. Because of this heterogeneity, to define a healer can be seen as problematic [26]. Still for clarification of the term, in this study, a healer, often also called “spiritual” healer, is defined as a person who exercises above all the practice of laying on of hands, prayers, and/or meditation while most importantly considering himself connected to a transcendent or spiritual power [28, 29]. We define transcendence as an experience beyond one's own self which may or may not be linked to religion or spirituality [30, 31]. Spirituality then is defined as the search for the connection to a transcendent reality which refers to the divine or supernatural beings (spirits, God, a higher power, or the higher self) [32, 33]. Today, healers in Germany who are medical professionals, “Heilpraktiker” (nonmedical practitioners with an education in complementary and alternative medicine = CAM), or healers without CAM or medical education can be found. Healers without CAM or medical background have various professional backgrounds and perform healing often as a side job. Little data exists in general about the healers' education in healing but can derive from various educations in spiritual healing offered, for example, by other healers [24]. By law, spiritual healing in Germany is permissible since 2004 for the purpose of activating self-healing powers and does not replace diagnosis or treatment by a physician or “Heilpraktiker” [26]. The conditions people consult healers about are mostly somatic or psychosomatic chronic and spiritual problems [24].

The aim of this study was to understand the growing field of contemporary healers and their clients in Germany and to learn about their subjective experiences, biographies, concepts, and motivations. To do so, we conducted a qualitative study integrating perspectives from medicine, medical anthropology, and religious studies. For the purpose of this paper, we focused on how healers and their clients describe and discuss their relationships. This discussion allows for a conceptualization of the healer-client relationship in German healing settings.

## 2. Materials and Methods

### 2.1. Study Design

A qualitative study based on semistructured interviews with healer and clients combined with participatory observation was conducted. To include different perspectives, the research team was interdisciplinary, composed of three physicians, two medical anthropologists, and one religious studies scholar who were all experienced qualitative researchers. The religious studies scholar had practiced healing; all the other researchers had no background in practicing spiritual healing. The whole team was involved in the development of the interview guidelines, recruitment process, data collection, and data analysis.

The interviews were designed to elicit information on biography; motives to consult a healer or to heal; expectations and explanations of the healing process; concepts of health, illness, and healing; experiences during and perceptions of the healing sessions; and the participants' perceptions of the effects and outcomes. The interview guide was used to support the interviewers and allowed flexibility to vary and deepen particular aspects of interest. After the first interviews, the interview guide was critically reviewed by the research team and improved accordingly. This paper focuses on the statements of healers and clients about their relationships.

### 2.2. Sample and Data Collection

Because the field of healers in Germany is very heterogeneous and not in general easy to access, a snowball sampling technique was used for recruitment [34]. The researchers used different ways to make contact with the healers. One way was to follow expert recommendations of various healer organizations. Attending conferences and workshops was another strategy to recruit healers and their clients into the study. Finally, recommendations and advertisements from healers themselves were used to get to know healers. Healers in rural and urban areas were included. Recruitment was aimed at including healers with medical education, “Heilpraktiker” (nonmedical CAM practitioners), and healers without any medical or CAM background. The clients were selected by the interviewed healers.

Both interviews and healing sessions were digitally recorded and then transcribed verbatim. Materials were pseudonymised. Written memos of the interviews and participatory observations by the researchers added further information on the setting, nonverbal expressions of the interviewees, and the researchers' subjective experiences.

### 2.3. Analysis

The data was analyzed based on a directed qualitative content analysis [35] with the computer program MAXQDA. Categories and codes were developed inductively from the data and deductively according to the themes of the structured interview guide and the new topics which arose during research. The analytic process was circular and triadic, meaning that new insights from the first data analysis were included in the subsequent data gathering and interpretation processes. The research team met every two to three months to discuss, reflect, and optimize the data collection and analysis. To improve quality and validity of the analysis and to ensure multidisciplinarity and intersubjectivity, all coded interviews were reviewed and recoded by another researcher of the team before further analysis. All data were discussed by the entire team according to the different disciplines and perspectives [36]. Thus, concepts from anthropological and religious studies related to our findings (e.g., concepts of embodiment and radical empathy) [37, 38] and medically important aspects, such as symptoms or outcomes [39], could be integrated.

### 2.4. Ethical Issues

Participants provided written informed consent and the study was approved by the ethics committee of Charité University Universitätsmedizin (EA1/238/10).

## 3. Results and Discussion

### 3.1. Results

In total, 15 healers and 16 clients were included in the study. The healers were 9 males and 6 females, with a mean age of 55 ± SD 7.9 years. One healer was a nurse, four healers were physicians, four were “Heilpraktiker” (nonmedical CAM practitioners), and six had no medical or CAM education. All healers worked full time or part time as spiritual healers for already many years. For example, one of the physicians had stopped practicing conventional medicine; the other three physicians practiced both, conventional medicine and spiritual healing, but never in combination and at the same time. The “Heilpraktiker” often combined spiritual healing with other CAM methods. The healing techniques mainly used by all healers were laying on of hands and/or prayer healing. The healers reported that healing talents can be inborn, developed, or acquired during life. Mostly, the experience of a powerful event or a crisis prompted their transformations into healers (all healers without CAM or medical education and half of the healers with CAM or medical background). This transformation often took many years and was often accompanied and supported by other spiritual healers and teachers. However, some of the interviewees (all with CAM or medical background) talked about becoming a healer out of sheer interest and learning to heal by attending courses in spiritual healing (for more details, see [38]).

Of the 16 clients, 13 were female and 3 were male and they were on average 56 ± 13.8 years old. Three clients were working in health care, two in the media industry, one as a lawyer, one client was unemployed, and four were pensioned. All included clients knew their healers already for a longer time, sometimes years, and had a trustful relationship with them. Reasons for consulting a spiritual healer were mostly chronic somatic problems (e.g., pain), serious illnesses (e.g., cancer), psychological problems (e.g., feeling depressed, stressed, exhausted, fearful, or in panic), or social problems. In addition, we conducted and analyzed eight participatory observations of healing sessions. The data collection took place at the healers' houses or offices (all participatory observations and most of the interviews), at the houses of the clients (some interviews with clients), and at the research facilities of the Charité (very few of the interviews with clients).

Throughout all interviews, the relationship between healer and client was characterized as a very special one, a relationship based on mutual respect and appreciation. The experienced relationship in the healing session was described by most healers and clients as a profound and “*unique*” contact. That contact was said to allow the sharing of emotions and visions and was often described as a spiritual experience. The personality of the healer was of high importance for the clients to build trust in the healer and in the sometimes challenging healing process. Moreover, the healers were frequently seen as role models for healing to occur and support for being spiritually connected. The clients, on the other hand, had to be generally willing to open up for this connection and to simultaneously take individual responsibility. Both healers and clients emphasized the importance of the healers' empathy.

#### 3.1.1. The Experience of Profundity and Uniqueness between Healer and Client

The “*unique*” relation between healer and client was said to start with the first encounter wherein a mutual understanding existed in being willing to engage in the healing process and to trust.

 D_H2_K1, client: “*Within the first contact it was already quiet pleasant. There was immediately such confidence … I think the chemistry has to be right … because you quite reveal yourself.*”

Healers and clients described their relationship as “*very deep*” and repeatedly used terms like* “energy flow,” “resonance,”* and* “fusion [Verschmelzung]”* to describe the close and intense healer-client association during healing sessions. Sometimes, this bond evoked images and feelings shared by the healer and her/his client:

 C_H2, healer: “*Often I have the same images that the people also have, not similar, but exactly the same images.*”
 D_H1, healer: “*I often experience his [the client's] problems in my body. That means if something hurts him somewhere … then I feel the same pain he feels*.”
 A_H3, healer: “*And there an exchange takes place … from the energy of the healer to the person. An exchange, an act of love … on an energetic level … you connect a lot with somebody … one flow together.*”

Repeatedly, healers and clients talked about* “being touched.” *That was meant not only in a physical way but also figuratively or metaphorically. One can be touched physically, emotionally, and spiritually. Mostly, the clients were first touched physically by the healer through the intuitive laying on of hands which subsequently was said to enable the “*energetic*” connection between them and could evoke various emotions in each of them. Sometimes, a healer's prayer “*touched*” (in the sense of reminded of) the client's memories from childhood and allowed a reconnection to his spirituality. Again this is said to happen mutually between healer and client.

 C_H2, healer: “*I would only touch people where I have the feeling it is right. … In ninety per cent [of cases] the patients say afterwards: you touched me exactly there where I thought I needed it.*”
 C_H2, healer: “*And many patients say, ‘the last time grandmother has prayed with me' … or your mother if you were ill, at your bedside, only for yourself. And that is exactly that what opens something up in the people … something very, very, very deep.*”
 C_H1_H2_K3, client: “*For me, it had to do with where I was touched, for example, if I were touched on the knee, I had the feeling as if … I'll call it - and I can't exactly define it - ‘healing energy' was flowing. … And when C_H2 touched my foot, for example, I have the feeling of the connection between the earth and the heavens, the connection simply to my faith!*”

#### 3.1.2. The Healers' Power to Connect to the Spiritual

Clients often seemed to be very impressed by their healers and saw them as ideals to strive for. They described their healers as powerful, knowledgeable, and wise. Due to the healers, they were reassured about what they believe in and hope for, but to which they had often lost contact in their normal lives. Thus, clients reported finding peace within themselves and a connection to a divine power during a healing session.

 A_H2_K1, client: “*She [A_H2] has … an unbelievable power … and then, things always just go much easier. When I am at home on my own … and want to do such things … then it isn't that easy anymore … I don't know what it is about this woman, she has a power which is unbelievable.*”
 C_H1_H2_K6, client: “*C_H2 has so much knowledge! … that I have the feeling … here you are in good hands. That is like a stairway to God.*”

The healers saw themselves mostly as mediums or* “channels,” *as intermediaries who try to act without intention and follow the guidance of a higher order or being. Healers saw themselves as persons in contact with their intuition and a spiritual power. Identifying as a channel enables the healer also to allow a profound association with the client in the healing session while still staying personally detached.

 D_H1, healer:* “A good healer is just somebody who leads the energy through ….”*
 A_H3, healer:* “you leave the personal level, our opinions, ideas and conditionings … and connect yourself … with the ocean of knowledge. You can call it also collective unconsciousness or the higher self. Call it as you want, if you want, call it God … and if you have a question, then the question pulls the answer … it does not come from a personal level, that is the decisive factor … It comes through you, you are a channel, and you receive the answer out of the ocean.”*

Often the healing session is experienced by the clients as a dream-like or trance-like state where the healer is seen as somebody guiding them through (see also our publication on perceived outcomes of spiritual healing and explanations in [39]).

 C_H1_H2_K6, client: “*it's a very deep relaxation. And then I see the light. Sometimes there are light frequencies and sometimes I have the feeling that Mother Mary [the Virgin Mary] is present … and somehow I then feel so much healing and protection*.”
 D_H3_K1, client:* “Dream images might come close [to explaining the experience], but it's more perhaps multi-dimensional impressions of one's own being that are revealing themselves … these facets of being, that show themselves across differentiated levels.”*

#### 3.1.3. The Importance of Empathy

The healer's empathy was very important for healers and clients alike. The term empathy, in this context, means something like giving one's full attention to the client, having an intuitive understanding of the client's personality and health history, and being present and open without having any prior judgments or intentions.

 C_H4, healer:* “… that I am there, just there and be at their [the client's] side, being a good friend, somebody who listens, who does not judge, who does not condemn.”*
 A_H3_K1, client:* “The therapeutic relationship is very important … but that is -in the end- … empathy … no judgment, no intention.”*

Many healers reported that they have this profound empathetic understanding for their clients because of their intuitive, subtle, or clairvoyant perception.

 B_H3, healer:* “Maybe … I have the special talent for an extraordinary understanding for the patients. Not only for the patients, their sorrows and needs, but also for the illnesses they have … what an illness does to a person.”*

Following this intuitive, empathic understanding, verbal communication was often not necessary for the healers.

 C_H1, healer:* “If somebody says nothing, you hear what he wants to say.”*

Mostly, healers would speak with the clients during the healing session only because the clients were used to it and would become irritated if the healers remained silent. At the same time, the conversation prior to and after the healing session was seen as important by the healers. Thus, the clients would have the opportunity to share experiences or to clarify questions which arose in the wake of the healing session and which could help the client to better understand what had happened.

#### 3.1.4. The Healers as Companions of Personally Responsible Clients

The healers saw themselves as* “companions”* and* “counselors”* in the clients' healing process. Thus, they tried to support their clients in activating and deploying their self-regulating forces. Because of the healers' own experiences of crisis, illness, and self-healing, the healers promoted their clients' hopes for healing and their belief in a solution for their problems. Despite the close relationship, the healers emphasized the importance of not influencing the client or building up dependency.

 A_H2, healer:* “I never would impose something on somebody.”*
 A_H3, healer:* “… actually, I accompany him there … and sometimes I push him a little bit.”*

Many healers and clients emphasized the meaning of individual responsibility. For the clients, it was important not to be patronized, to be able to decide freely, and not to be told what they should or needed to do. Often, clients had already demonstrated independence in their choice of healer and often enough finding the right healer for them had already taken a long time.

 A_H3_K1, client:* “Eventually I find the therapists, and then we … look together … for the way. … but, in principle … I am the first one who initiates it, I am the last who decides it, because I must also carry the consequences.”*
 C_H1_H2_K3, client:* “I don't feel particularly influenced … like of a [psycho-] therapist … Here I am not told what to do as homework.”*

According to the healers, the client's personal responsibility is an essential aspect of the healing. From the healer's perspective, healing is possible only if the client is ready to assume the responsibility for himself. Personal responsibility, in this context, would not mean that the client was to be blamed for his problems and illnesses. Rather, personal responsibility refers to the strength and courage to overcome fears and to actively participate in the healing process. Often people would not want to assume responsibility but would prefer to be told what to do in their lives. Many healers would not encourage such an imbalance in power but emphasized reciprocal responsibilities.

 D_H3, healer:* “… the willingness of some people to hand over the responsibility is just very big and therefore it needs strength [of the healer] to say, no, I don't take the responsibility, I leave it with you ….”*

### 3.2. Discussion

The relationship between healers and clients during the encounters was described as profound and unique, wherein healer and client shared emotions and sensations and connected to a transcendent source. The personality of the healer supported the clients' hopes for healing and helped the clients to (re)connect to their own spirituality. The healers' empathy was emphasized by both healer and clients and was explained by the healers as a subtle, intuitive understanding of the other. The relationship was seen as partner-based. The healers were said to act as a companion to the client in his healing process. Clients had to be open for this profound connection to occur and at the same time to be willing to assume personal responsibility.

#### 3.2.1. Strengths and Limitations

One strength of this qualitative study is the inclusion of a range of spiritual healers and their clients from different settings (rural, city), (religious) traditions, healing techniques, and professions. Another strength of this study is the interdisciplinarity in the research team, which included scholars in medicine, anthropology, and religious studies. That diversity permitted different approaches in the field. The same diversity contributed to various perspectives in the analysis. The interdisciplinary teamwork led to complex and time-consuming processes and a practicable balance had to be found between all aspects of the extensive data material and the interdisciplinary experts' assessments.

A limitation of this study is the selection for our sample as clients were included in this study by their healers. It is likely that they have chosen clients with whom they work well. Therefore, the reported outcomes and explanations might present a picture that describes only successful healing sessions and stories of clients that improved during the healing process. This means that our results possibly reflect the more positive aspects of healing processes and preclude failed stories. Furthermore our interviews took place* a posteriori *when the process of healing had already occurred and healers and their clients might have constructed new meaning and shared beliefs together. Furthermore, it should be considered that, basically in every interview study, words can be understood only as an abstraction of experience. Also in this study the interviewees reported repeatedly that it was difficult to find words for their experiences, feelings, and concepts concerning a spiritual experience.

#### 3.2.2. The Triangular Relationship between Healer, Client, and the Transcendent

The statements of healers and clients about the importance of the relationship between them correlate to studies about doctor-patient relationships and therapeutic relationships in psychotherapy as described in the Introduction [1–4, 6–10]. Still, the relationship between healers and clients differs from doctor-patient and from psychotherapeutic relationships. The major difference is an additional connection to the transcendent in the healer-client relationship, from an etic perspective so as to say a triangular relationship of healer, client, and a transcendent source. The healer sees himself therein as a channel which allows an intuitive, subtle, or even clairvoyant perception. The clients experience this as being* “deeply touched”* in a* “unique”* way. They were felt, seen, and understood by the healer and they spiritually (re)connected. That experience was characterized as very meaningful to the clients and was not found easily in normal life or in their experiences with conventional medicine. The connection to the spiritual/transcendent is said to directly influence the empathetic understanding of the healer and will be discussed in the following section.

#### 3.2.3. Empathy and Its Relation to “Fusion [Verschmelzung]”

Empathy was of high importance for the interviewees and encompassed subtle perception, awareness, presence, nonjudgment, and the company of the healer without imposing influence. Even though the term* empathy* is nowadays an inherent part of clinical and psychological research, historically there has been much debate about the origin of the term and its meaning. Interestingly, explanations of the German term “Einfühlung” (which was subsequently translated as* empathy* [40]) relate to frequent statements of the healers about* “fusion [Verschmelzung].”*

 
* It is the fact that the contrast between myself and the object disappears.* (Lipps in [40]: 154-155)

Still these first debates about “Einfühlung”/empathy in the nineteenth century in Germany were philosophical rather than spiritual and first began in art and somewhat later infiltrated psychology. A central question was how it is possible to know what somebody else feels. It has been argued that, by reproducing or reexperiencing another's experience, it would be possible to get a higher understanding of the other [40]. This notion is reflected in healers' statements regarding the healer's ability to feel what his client feels.

 
* … the perception and comprehension of … another individual … does not mean that we see it or apprehend it by means of the senses. We cannot do that …. We can only experience this kind of thing in ourselves.* (Lipps in [40]: 156)

While there are still debates about the definition [6, 41], today in the context of clinical medicine, empathy is mainly defined as* “the ability (1) to understand the patient's situation, perspective and feeling …, (2) to communicate that understanding …, (3) to act on that understanding with the patient …” *[42]: 9. That definition of clinical empathy does not necessarily mean to feel exactly what the patient does. Indeed, such a phenomenon could evoke an overidentification with the patient and a loss of professional boundaries [43]. The clinical medicine definition differs from how empathy is understood by the healers in our study. Most healers reported feeling exactly what their clients felt but were not attached to it; rather the healer acted as a channel through which these sensations passed. Moreover, the healer recognized that in his/her role as a channel, he/she connected to a spiritual power. Still our data leaves as an open question whether or not the client conversely feels what the healer feels. It rather seems that, in the healing session, both healer and client experience “fusion,” in which the healer helps the client to access a transcendent source and the healer himself perceives the problems of the client because of being connected to the transcendent. Koss-Chioino talks in this regard of “radical empathy,” meaning that, by connecting to a transcendent source, the healer can be radically empathetic with his clients [17]. Based on that concept and correlated to the shared sensations in a healing session reported in our study, Jeserich speaks of “radical body empathy” because radical empathy also seems to be embodied empathy [37].

#### 3.2.4. Participatory Healer-Client Relationship

The importance of the personality of the healer is emphasized in this study and correlates with various anthropological studies about healers and their patients [11–16]. In our study, the healers either inherited power, wisdom, and their healing abilities or developed these attributes through their personal experience of surrendering crisis or illness. The personality of the healer supported the clients' trust in the healer, hopes for healing, and (re)connection to their own spirituality. Salutogenic factors could therefore be enhanced and the clients often learned to give a different meaning to their lives, problems, and illnesses [22, 39]. In this study, healers did not emphasize their power but rather their relationships with clients based on notions of partnership and being on an equal footing. That might contradict a preconception of the healer as a role model with the power to connect to the transcendent, which on his own the client cannot achieve. Still most of the healers describe themselves more as coaches that show clients a path that the healer has already taken in his past. Clients feel supported rather than told what to do.

This partner-like approach of contemporary healers in Germany is possibly influenced by the current general discussion about paternalism versus partnership in health care [9, 44]. Partnership relationships might be easier to realize in the healer setting; healing encounters typically last one hour or longer and are generally free from hierarchically structured organizations, unlike in conventional medical systems. Still it must be also taken into account that there might be clients who prefer a paternalistic setting, for example, the healer providing the access to the spiritual for them.

The client had to be willing to open up to this relationship and at the same time to be willing to assume personal responsibility. Critically, it should be noted here that the close relationship between healers and their clients could contradict independence, noninfluencing, and individual responsibility. Even if not expressed in our data, we must recognize that the healer setting is not immune from fostering dependence and that abuses of power may occur [45, 46]. Possibly that is exactly the reason why the healers stressed the individual responsibility of the clients. Dependency in health care systems and especially in mental health care and psychotherapy is well known and has to be handled carefully by the therapists. In the earliest stages of psychotherapy, dependency can be an important phase in which a close working alliance between therapist and patient based on trust and respect is established. But further along in the process, an increasing independence is needed for the patient to be able to handle his life on his own [47]. That might be the same in the healer-client relationship as the interviewees expected their healers to have high ethical standards and the ability to self-reflect.

#### 3.2.5. The Spiritual/Transcendent in Health Care

The findings of our study also show the importance of spiritual questions related to health care. It is not only the relation to the healer that matters for the client, but also the client's relationship to the spiritual/transcendent. In this sense, Cox proposes that the existing biopsychosocial model should be extended to a “body-mind-spirit paradigm” in individual therapies [48]. He sees in this paradigm a necessary integration of science, psychology, spirituality, and the quest for meaning in life. In conventional medicine, with an emphasis on technology, interpersonal relationships often suffer [9] and partly it is requested that the doctor should relate back to his role as a healer who attends mind, body, and spirit [49]. Because of conventional medicine's technically orientated setting, combined with a high work load and little time for communicating with patients, the ideal of a physician who attends to body, mind, and spirit might be difficult to realize [50]. In psychotherapy, however, a trend toward spiritually orientated psychotherapists is noticeable [51–53].

## 4. Conclusions

The results of this study stress the importance of and potential in the relationship between healer and client. In contrast to doctor-patient and therapeutic relationships, central to healer-client relationships were the shared experience in healing sessions and the triangular relation between client, healer, and the transcendent. The healers see themselves therein as a channel to the spiritual/transcendent which would allow them a subtle empathetic understanding of the client while staying personally detached. Furthermore, the personality and a partner-like attitude of the healer supported the client's efforts to give a different meaning to his problems and his life, to (re)connect to his spirituality, and to assume personal responsibility. In further studies, the question of how spirituality may be integrated in health care and how the healer-client relationships develop over time in the healing process could be of interest.
